# Impact observations of asteroid Dimorphos via Light Italian CubeSat for imaging of asteroids (LICIACube)

**DOI:** 10.1038/s41467-023-38705-0

**Published:** 2023-05-26

**Authors:** Elisabetta Dotto, Angelo Zinzi

**Affiliations:** 1 INAF-Osservatorio Astronomico di Roma, Via Frascati 33 00078 Monte Porzio Catone (Roma), Italy; 2 ASI, SSDC, Via del Politecnico s.n.c., 00133 Roma, Italy

**Keywords:** Astronomical instrumentation, Asteroids, comets and Kuiper belt

## Abstract

On September 26^th^ 2022, LICIACube monitored Double Asteroid Redirection Test (DART) mission impact on asteroid Dimorphos, which is the smaller component of a binary asteroid system. These close observations revealed the impact ejecta features of the first planetary defence test with a kinetic impactor.

## LICIACube as a cubesat for planetary defence

LICIACube, constituted by a CubeSat with dimensions of 10x20x30cm, is the first Italian deep space satellite managed by the Italian Space Agency (ASI)^1^. This class of small satellites gets its name from being based on a form factor consisting of 10 cm cubes. Thanks to their small size and great versatility, CubeSats have a wide variety of applications and they are now starting to be of interest also in solar system exploration. Such examples include, Mars Cube One (MarCO), launched with the NASA Interior Exploration using Seismic Investigations (InSight) Mars lander to support the communications with the Earth during the entry, descent and landing phases of the probe^2^, and the ten CubeSats deployed with Artemis I mission, designed to study the surface of the Moon and its radiation environment, but also for Near Earth Object (NEO) exploration and for technological aims (e.g., Argomoon^3^).

Despite its small size and mass (less than 14 kg), LICIACube hosted two different cameras, -Liciacube Explorer Imaging for Asteroid (LEIA) designed to manage both CubeSat operations and obtain scientific images, and -Liciacube Unit Key Explorer (LUKE) able to acquire images at different wavelengths (i.e., red, green and blue colours) allowing scientists to investigate the material properties^1^. The design and construction of LICIACube was a real technological challenge as it had fulfil both payload requirements (i.e., small size and mass) to be housed in the DART spacecraft^4^, and at the same time to be -able to fulfil operational and scientific tasks: recognise its target, perform orbital manoeuvres, navigation, acquire scientific images and witness the DART impact on Dimorphos.

The NASA mission DART successfully performed the first kinetic experiment for Planetary Defence^5^ by impacting on Dimorphos, the 160 m satellite of the binary Near Earth Asteroid (65803) Didymos, with the aim of deflecting it on its motion around the central body. LICIACube travelled with DART, as a piggyback, during 291 days of cruise. Released on September 11^th^ 2022, LICIACube started its navigation towards the target Dimorphos, to observe the DART impact and to obtain scientific images of its aftermath. The DART impact transferred a momentum to Dimorphos^6^, reduced the orbital energy of Dimorphos, decreasing the semi-major axis of its orbit and its orbital period around Didymos, hence resulting in an increased orbital speed^7^. The study and modelling of this effect, combined with the deformation of Dimorphos after the impact, could give rise to a series of dynamic effects such as the tidal evolution of the system coupled to non-gravitational effects^8^. In the context of planetary defence, one of the major goals of the whole mission is to determine the variation of the dynamics of the system, and one of the fundamental inputs are the speeds of the particles ejected by the impact(impact ejecta). Therefore, the LICIACube images are of pivotal importance to their calculation, providing complementary information to that obtained from Earth^7^ and HST observations^9^. The reconstruction of the ejecta dynamics at different time and size scales needs to be coupled with an accurate modelling of the dynamics of the Didymos-Dimorphos binary system which is, in turn, a laboratory for orbital dynamics (orbital perturbations, tides, chaos, …) and asteroid formation models.

## The LICIACube flyby

LICIACube performed its flyby, reaching the minimum distance of about 58 km from Dimorphos, roughly 168 s after the DART impact. The impact of DART was testified as a change of brightness of the target, seen by LEIA. LUKE started to acquire images 29 s after the impact and both the instruments observed the target and the evolution of the impact effects up to 320 s after the nominal impact time. These LICIACube images will be crucial, in combination with those acquired by the camera onboard DART, to reconstruct the 3D shape models of both Didymos and Dimorphos. This effort is fundamental for the correct modelling of the dynamics of the binary system^10^.

**Fig. 1:**
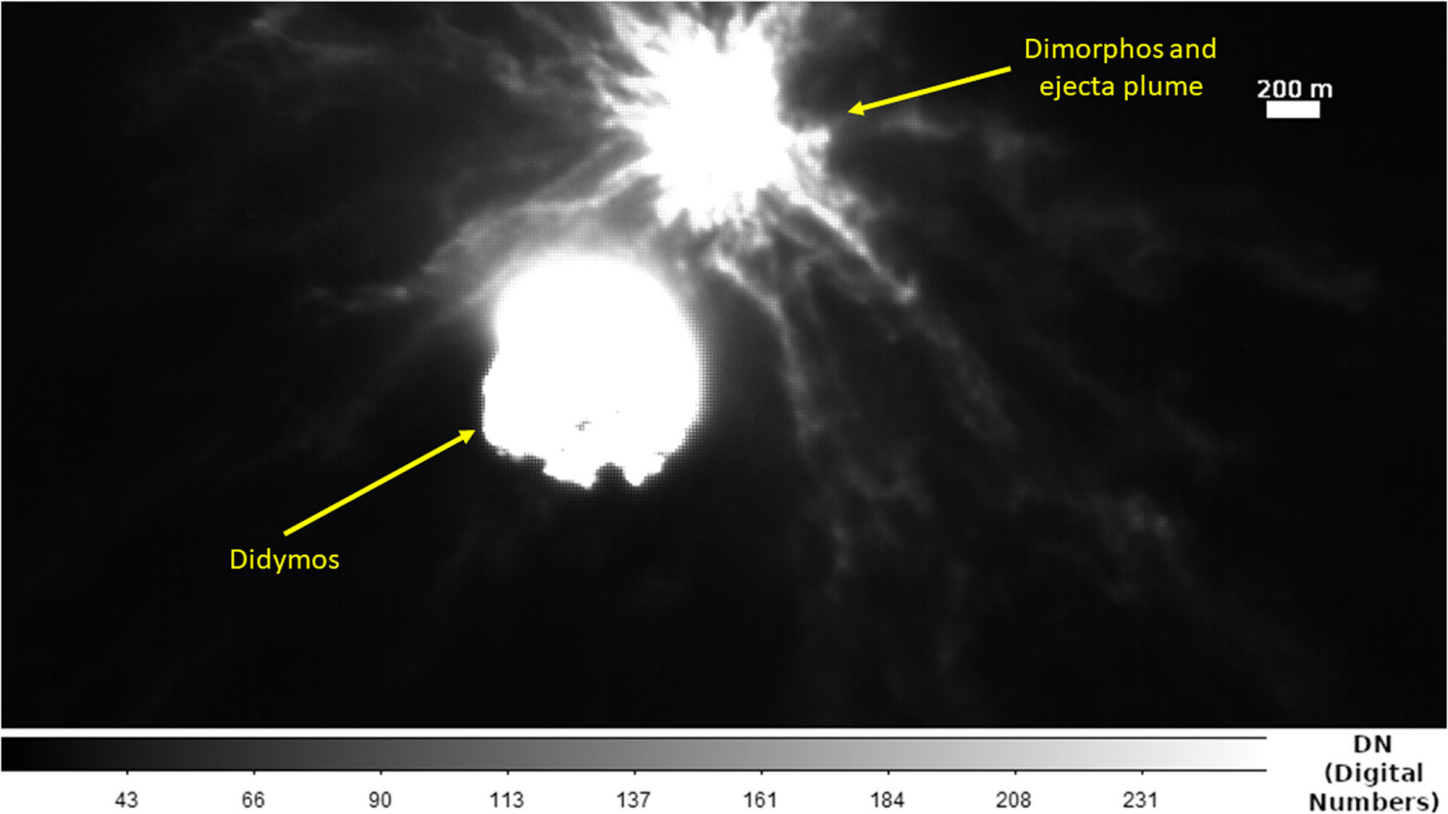
LICIACube LEIA image of the Didymos-Dimorphos system after the DART impact. The structure of the ejecta plume is visible from the imaging at 82 km distance from Dimorphos (Credits ASI/NASA).

From its privileged position LICIACube was able to acquire images of a very extended plume that completely masked the impact site on Dimorphos (Fig. 1). The LICIACube images (available in: https://www.ssdc.asi.it/liciacube/) clearly show a dust cone and boulders ejected from the surface of Dimorphos, as a result of the impact; the plume exhibits an irregular pattern with filaments and inhomogeneous regions.

The images acquired by LICIACube during the flyby are presently being analysed to investigate the structure of Dimorphos using ejecta cone aperture, its structure, the distribution of velocities, and the study of its morphology at the initial phase of its expansion.

LICIACube has also acquired images of the unaltered (not impacted) side of Dimorphos in three colours (red, green and blue). The colour distributions in specific locations of the plume and on the surfaces of Didymos and Dimorphos, allow to constrain their nature and composition as well as the degree of alteration of the asteroid surface, thanks to the great opportunity offered by the excavation of unaltered material from the subsurface of the Dimorphos^11^. Possible differences in colour between Didymos and Dimorphos, can be also related to the origin of the system and could allow us to investigate in detail how binary asteroids form and evolve^12^.

## Future aspects

Albeit traditional planetary exploration missions have very different cost ranges, depending upon duration, size, scientific instrumentation, LICIACube can be considered a “low cost mission”, as its development costs are roughly one order of magnitude lower than the ones afforded for similar missions’ destination and objectives, through spacecrafts of traditional size. Due to their quick and low-cost development Cubesats represent a turning point in planetary exploration as well as in planetary defence. Their use is obviously extremely ambitious and challenging but, at the same time, provides a high scientific and technological return, validating procedures in space performed with miniaturised instruments. LICIACube, after MarCO^2^, has been another successful demonstration of a CubeSat operating in deep space and it set a record for a spacecraft in its size class, being the first CubeSat able to perform orbit manoeuvres, point the target, acquire more than 400 images of a unique event, and send the obtained data directly to Earth.

The planetary defence scientific and political community will certainly deeply benefit from the first planetary defence test obtained from the DART mission. Key experiences by LICIACube that would support future missions include the ability to adequately exploit the limited on-board resources, maintaining a high-level of capabilities and performances, by complementing missions usually assigned to larger spacecrafts. While there are several CubeSat mission concepts under study (e.g., Ref. ^13^ and Ref. ^14^), an idea for future CubeSats applications in planetary defence could be a low-cost planetary exploration space missions dedicated to study asteroids that are potentially hazardous to Earth.

DART mission successfully tested one of the hazard mitigation techniques, kinetic impactor, and demonstrated the technological readiness. LICIACube’s successful operations led to the imaging of such a unique event from the vicinity of the impact scenario. The next planetary defence mission hosting CubeSats will be the Hera mission from European Space Agency (ESA)^13^. Together with DART, Hera is part of the international Asteroid Impact & Deflection Assessment (AIDA) collaboration and it will be launched in 2024 to rendez-vous the same binary asteroid (Didymos-Dimorphos) system in late 2026. Hera mission instrumentation includes two different CubeSats: Juventas, which will be the first cubesat with a low-frequency radar to scan the interior structure of an asteroid, and Milani, which will perform multi-wavelength (from visible to short wave infra-red, 500–2500 nm) imaging of the Didymos-Dimorphos system following DART impact. The data obtained by Hera mission will then be combined with DART and LICIACube data for the most comprehensive characterisation of a binary asteroid system and for the investigation of the potential changes due to DART impact.
