# Author Correction: Impact observations of asteroid Dimorphos via Light Italian CubeSat for imaging of asteroids (LICIACube)

**DOI:** 10.1038/s41467-023-39145-6

**Published:** 2023-06-16

**Authors:** Elisabetta Dotto, Angelo Zinzi

**Affiliations:** 1 INAF-Osservatorio Astronomico di Roma, Via Frascati 33 00078 Monte Porzio Catone (Roma), Italy; 2 ASI, SSDC, Via del Politecnico s.n.c., 00133 Roma, Italy

**Keywords:** Astronomical instrumentation, Asteroids, comets and Kuiper belt

Correction to: *Nature Communications* 10.1038/s41467-023-38705-0, published online 26 May 2023

The original version of this Article contained an error in the spelling of the authors Elisabetta Dotto and Angelo Zinzi, which was incorrectly transposed as Dotto Elisabetta and Zinzi Angelo. This has now been corrected in both the PDF and HTML versions of the Article.

